# Association of smoking, physical activity, and dietary habits with socioeconomic variables: a cross-sectional study in adults on both sides of the Hungarian-Romanian border

**DOI:** 10.1186/1471-2458-12-60

**Published:** 2012-01-20

**Authors:** Erika Nédó, Edit Paulik

**Affiliations:** 1 Réthy Pál Hospital and Policlinic, Békéscsaba, Hungary, H-5600 Békéscsaba, Gyulai út 18; 2 Department of Public Health, Faculty of Medicine, University of Szeged, Szeged, Hungary, H-6720 Szeged, Dóm tér 10

## Abstract

**Background:**

The association between socioeconomic status and health-related behaviours has been clarified in several epidemiological studies. The aim of this study was to reveal the socioeconomic differences in health-related behaviours and in nutritional status of Hungarian and Romanian citizens living on both sides of the border.

**Methods:**

A cross-sectional study based on interviewer-administered questionnaires was conducted on both sides of the Hungarian-Romanian border. The survey was completed by 1, 099 Hungarians (Hu) and 852 Romanians (Ro) aged 18 years and over; the overall participation rate was 92.9%. Demographic and socioeconomic factors, health-related behaviours (smoking, dietary habits and physical activity), body weight and height were recorded. All analyses were performed separately for Hungarians and Romanians. Simple descriptive statistics and logistic regression models were used to measure the associations between socioeconomic status and behaviour, as well as obesity.

**Results:**

The prevalence of smoking was similar in Hungarians and Romanians (33.2% and 36.4%). The frequency of "unhealthy diet" was 70.6% in Hungarians and 75.2% in Romanians. Physical inactivity was more prevalent in Romanians (73.2%) than in Hungarians (32.0%), while the prevalence of obesity was higher in Hungarians (22.0%) than in Romanians (16.5%). Based on the univariate logistic regression models the risk of smoking was higher among those with medium educational level (ORHu = 1.66) and poor financial conditions (ORHu = 3.13) in Hungarians. The risk of unhealthy diet was higher among the low educated (ORHu = 1.77; ORRo = 7.91) and among those with poor financial conditions (ORHu = 2.05; ORRo = 4.25). None of the socioeconomic factors was associated with leisure time physical inactivity. In the multivariate models obesity was associated with medium level of education in Hungarians, and with unhealthy diet in Romanians (ORRo = 2.10). Physically inactive Hungarians were more (ORHu = 1.74), whereas inactive Romanians were less (ORRo = 0.64) likely to be obese than physically active people from the same country.

**Conclusions:**

The present study shows that socioeconomic status is associated with health-related behaviours in a small area of Hungary and Romania. The results highlight the need for developing interventional strategies, focusing more on people in lower socioeconomic status, in order to reduce the existing inequalities in health and health-related behaviours.

## Background

There are large variations in the prevalence of chronic diseases among European countries with regard to socioeconomic differences [1]. The diseases in question, such as heart disease, stroke or diabetes mellitus, have a multifactorial aetiology including individual characteristics and health protective factors, together with social, economic and environmental determinants [2].

The association of socioeconomic status (SES) with health and health-related behaviours (e.g. smoking, diet and physical activity) has been supported by several epidemiological studies. Smoking is more frequent among lower educated people in developed countries and especially common among the socioeconomically disadvantaged [3-8]. Therefore, tobacco smoking is one of the most important determinants of social inequalities in health in the developed world nowadays [9]. Unfavourable socioeconomic status is associated not only with tobacco smoking, but with physical inactivity and obesity. Currently, sedentary lifestyle is a severe "epidemic" in the European Union countries [10,11]. Physical activity has been found to be associated with SES: those with higher educational level or professionals were more likely to be active but maximum at moderate intensity [12]. Education proved to be associated with indicators of a healthy diet in Norway [13]. People across Europe with lower SES consume nutrients from a less diverse food base: they eat monotonous diets with little variety [12]. The connection between socioeconomic and lifestyle factors with overweight and obesity has likewise been confirmed in adult populations [14-16].

The inequalities in health and health-related behaviours can be detected between the Eastern and Western parts of Europe. This 'East-West Health Gap', that is one of the biggest challenges today, is the result of the additive effect of socioeconomic factors and widespread health-damaging behaviours [2,12,17-19]. This problem is especially recognisable in Romania that joined the European Union in 2007 but also in Hungary (EU member since 2004). The health state of the population of Hungary and Romania shows similar trends in mortality [20-24]. The general state of health of Hungarians and Romanians is worse than justified by the level of economic development. Life expectancy both in Hungary and in Romania is among the lowest in Europe [20,24,25]. Furthermore, large variations of life expectancy can be found in different parts of the countries. In case of Hungary, the life chances in the Eastern part of the country including the counties situated on the Hungarian-Romanian border are a great deal worse than that of the population in the Western part of Hungary [26]. There are also regional disparities within Romania as regards the health state of the population, e.g. the variation in life expectancy at the county level has shown differences in several areas: the highest is in the counties in the central part of the country, whereas the lower is in the counties in the northern and western parts including the counties on the Hungarian-Romanian border [22]. On the one hand, there are the similarities in health and geographic conditions and historic events in the neighbouring areas of Romania and Hungary on both sides of the border; on the other hand, there are no data on the similarities and differences in factors influencing health and their relationship with certain demographic and socioeconomic characteristics of the populations in question. During the economic transition from centrally planned to free market economy, health promotion programmes require information about the population's current health situation. Additionally, today there is an exceptional opportunity for both parties (Hungary and Romania) to elaborate common research and project as members of the Danube-Kris-Mures-Tisa Euroregion, and to establish programs aiming at changing the lifestyle of the population in the region by developing cooperation between the universities of Arad and Szeged. These facts motivated us to perform the present study.

Thus the aim of our research was to study the socioeconomic differences in health-related behaviours and in nutritional status of Hungarian and Romanian citizens living on both sides of the border. After describing and comparing the prevalence of health-related behaviours in the two countries, we wanted to differentiate between them according to the demographic and socioeconomic characteristics from the point of health-related behaviours. We wished to describe the characteristics of the Hungarians and Romanians following unhealthy behaviours. Based on these results we intended to define the target populations of our interventions to promote health among the people living in the two countries so as to support cooperation and development of cross-border community based health promotion programmes on both sides of the Hungarian-Romanian border.

## Methods

### Population

A population-based, cross-sectional health survey was conducted on both sides of the Hungarian-Romanian border, in February to June 2007.

A two-stage sampling was used. In the first stage of the sampling, the settlements on both sides of the Hungarian-Romanian border were selected. In Hungary, a small area of County Békés, including six settlements (towns and villages), was chosen. Another six settlements in County Arad, with similar characteristics in geographical location and population size, were chosen in Romania. Selecting the settlements for the research was not performed randomly as we involved each settlement from the affected small area in Hungary. Considering the fact that in Romania there are no small areas but mainly larger counties with a higher number of population, we targeted on selecting certain Romanian settlements (matched the settlements) having similar characteristics to the Hungarian settlements involved into the research. In the second stage, a sample stratified by age and sex was selected randomly from the Hungarian and Romanian citizens aged 18 and over (the mean age of the subjects was 48.23 years [SD 18.49; range 18-94] for Hungarians and 47.93 years [SD 17.72; range 18-90] for Romanians), making use of the local registries. The number of persons picked was proportionate to the population size of the settlements involved in the study. The overall sample of 2, 100 people (1, 200 Hungarians and 900 Romanians) represented approx. 2% of the target population. The survey was completed by 1, 099 Hungarians and 852 Romanians giving the overall participation rate of 92.9% (91.6% for Hungarians - 92.6% for males and 90.7 for females; 94.7% for Romanians - 94.9% for males and 94.4% for females.).

### Measures

The survey was based on interviewer-administered questionnaires pre-tested on 20 adult persons. Local family practitioners' assistants and midwifes were employed as interviewers after adequate training. Answering the questions was voluntary and anonymous.

Age, gender, educational level and financial condition were studied as demographic and socioeconomic factors. Educational level was categorized into three classes: low (no school, or primary school only), medium (vocational or secondary school) and high (college or university). Self-perception of financial conditions was based on the following question: "How do you evaluate your financial situation?" The five-point Likert scale - (1) very poor, (2) poor, (3) acceptable, (4) good and (5) very good - was used for the evaluation of self-perceived financial conditions. Because of the low frequency of "very good" and "very poor", the answers were grouped into three categories, "good" (very good and good), "acceptable" and "poor" (very poor and poor), during the course of the assessment.

Body mass index (BMI) was calculated from self-reported body mass (kg) and body height (m) and expressed in kg/m^2^. According to the recommendations of the World Health Organization [27], BMI was grouped into four categories as follows: underweight (< 18.5 kg/m^2^), normal weight (18.5-24.9 kg/m^2^), overweight (25.0-29.9 kg/m^2^) and obese (≥ 30.0 kg/m^2^). For the purpose of logistic regression BMI-groups were dichotomized as "obesity" (BMI ≥ 30.0 kg/m^2^) and "no obesity" (BMI < 30.0 kg/m^2^).

The questionnaire comprised questions on health-related behaviours such as smoking, dietary habits and physical activity.

Smoking status was assessed by the question: "Do you smoke?" with the options 'No, I have never smoked regularly', 'No, I have stopped smoking', 'Yes, occasionally', and 'Yes, daily'; the same question with similar options was used in several previous studies [5-7,28]. Smoking status of the respondents was described as never smokers, ex-smokers or current smokers (smoking daily or occasionally) at the time they were interviewed. For the purpose of analysis, smoking status was dichotomized as 'smokers' including current smokers, and as 'non-smokers' including ex-smokers and never smokers.

Dietary habits were evaluated on the basis of three questions about the frequency of fresh fruit consumption, fresh vegetable consumption and the kind of the fat (vegetable or animal origin) used for cooking. Respondents were asked e.g. how often they had eaten fresh fruit during the past month with the following options: 'daily, several times', 'at least once a day', '2 to 3 times a week', 'once a week', 'less than once a week' or 'never'. These questions were also used as indicators of healthy diet in the Hungarian "National Health Interview Survey" in 2000 [29]. Diet-related questions used in this survey were compiled by workers of the National Institute for Health Promotion according to the recommendations of the WHO and in accordance with the Hungarian Gallup Organization based on previous Hungarian findings [29]. On processing the data, these responses were converted into dichotomous variables, the consumption being recorded as "daily" if the answer was ' daily, several times' or 'at least once a day', while all other options were classified as "occasionally or never". In the final analysis, "healthy diet" was recorded when "daily" consumption of fruits, "daily" consumption of vegetables as well as the use of vegetable oil for cooking were recorded, and all the others were categorized as "unhealthy diet".

Subjects were asked to report on their physical activities. Regular participation in competitive sports and leisure-time physical activity was measured. Primarily, those who participated in competitive sports were regarded as physically active persons. People who did not pursue any competitive sport were asked about the regularity of their leisure time physical activity: "How often did you do the following forms of activity (running, swimming, gymnastics or using fitness machines, at least 20 minutes walking, bicycling and gardening) in the last year?" The options were the following: once a day, several times per week, once a week, several times per month, once a month, less than once a month and never. Those who participated in any form of exercise for less than several times per week (once a week, several times per month, etc.) were regarded as "physically inactive". Physical activity was measured in accordance with the recommendations of WHO and the EU, and questions were set according to the ones used in the Hungarian "National Health Interview Survey" [30-32]; the group of questions had already been used but had not been validated.

The study protocol was approved by the Regional and Institutional Human Medical Biological Research Ethics Committee of the University of Szeged (No. 118/2006.). Written informed consent was obtained from each participant of the study.

### Statistical analysis

All analyses were performed separately for Hungarians and Romanians. Simple descriptive statistics were used to describe the overall characteristics of the sample. Differences between Hungarians and Romanians were tested by Pearson's chi-square test. Univariate and multivariate logistic regression models were used to assess the effect of demographic (age, gender) and socioeconomic factors (educational level, financial conditions) on health-related behaviours (smoking, unhealthy diet and physical inactivity) and to reveal the associations between obesity and socioeconomic factors and health-related behaviours in two models. The odds for obesity were adjusted for age, gender, educational level and self-perceived financial conditions in Model 1, and for age, gender, educational level, self-perceived financial conditions, smoking status, dietary habits and leisure time physical activity in Model 2.

Age in years was introduced as a continuous variable and all other independent factors were included as categorical variables into the logistic regression models. The results are presented as odds ratios (OR) and 95% confidence intervals (95% CI). Statistical analysis was performed with the SPSS version 13.0.

## Results

The characteristics of the Hungarian and Romanian study population are presented in Table 1. Age and gender distribution was similar. Regarding education and financial conditions, however, there were differences: the rate of those with low education was higher among Romanians, whereas good financial conditions were more prevalent among Romanians than Hungarians. The prevalence of smoking was similar in Hungarians and Romanians (33.2% and 36.4%). The frequency of "daily" fruit and vegetable consumption was lower in Romanians, but regarding the kind of fat used for cooking, no difference was found. The frequency of "unhealthy diet" (as defined in the Methods) was 70.6% in Hungarians and 75.2% in Romanians. The frequency of leisure time physical inactivity was more than twice as high in the Romanian (73.2%) as in the Hungarian (32.0%) study population. Considering BMI, the prevalence of obesity was 22.0% in Hungarians (Hu), and 16.5% in Romanians (Ro).

**Table 1:** Demographic, socioeconomic characteristics, body mass index and health-related behaviours of the sample by nations

Variables	Hungarians (N = 1099)		Romanians (N = 852)		P-value*
	n	%	n	%	
Age-groups (yrs)					0.866
18-34	316	28.8	239	28.1	
35-54	383	34.8	293	34.4	
55 and over	400	36.4	320	37.6	
Gender					0.652
Females	575	52.3	437	51.3	
Males	524	47.7	415	48.7	
Educational level					< 0.001
High	197	17.9	147	17.3	
Medium	550	50.0	310	36.4	
Low	352	32.0	395	46.4	
Self-perceived financial conditions					< 0.001
Good	184	16.7	282	33.1	
Acceptable	634	57.7	432	50.7	
Poor	281	25.6	138	16.2	
BMI					0.006
≥ 30.0 kg/m^2^	242	22.0	141	16.5	
= 25.0-29.9 kg/m^2^	396	36.0	356	41.8	
= 18.5-24.9 kg/m^2^	429	39.0	336	39.4	
< 18.5 kg/m^2^	32	2.9	19	2.2	
Smoking status					0.144
Non-smokers	734	66.8	542	63.6	
Current smokers	365	33.2	310	36.4	
Fruit consumption					< 0.001
Daily	638	58.1	347	40.7	
Occasionally or never	461	41.9	505	59.3	
Vegetable consumption					< 0.001
Daily	471	42.9	282	33.1	
Occasionally or never	628	57.1	570	66.9	
Fat used for cooking					0.619
Vegetable oil	769	30.0	605	29.0	
Animal fat	330	70.0	247	71.0	
Leisure time physical activity					< 0.001
Active	747	68.0	228	26.8	
Inactive	352	32.0	624	73.2	

*Results of Pearson's chi-square test, BMI: body mass index

Tables 2 and 3 present the results of univariate logistic regression models. Based on these analyses, the risk of smoking decreased by age expressed in years (ORHu = 0.96, 95% CI: 0.96-0.97; ORRo = 0.98, 95% CI: 0.97-0.98), and males were more likely to smoke than females (ORHu = 2.29, 95% CI: 1.78-2.97; ORRo = 2.89, 2.16-3.86) both in Hungarians and Romanians. Hungarians with medium educational level (ORHu = 1.66, 95% CI: 1.16-2.38) and with poor financial conditions (ORHu = 3.13, 95% CI: 2.06-4.74) were more likely to smoke compared to those who were high educated or living in good financial conditions (Table 2). The risk of unhealthy diet was higher among the elderly (ORRo = 1.04, 95% CI: 1.03-1.05) in Romanians, but there was no association between the consumption of unhealthy diet and age in Hungarians. The association between the consumption of unhealthy diet and gender or socioeconomic indicators was similar in Hungarians and Romanians: the risk was higher among males (ORHu = 2.22, 95% CI: 1.69-2.90; ORRo = 1.75, 95% CI: 1.27-2.40), the low educated (ORHu = 1.77, 95% CI: 1.21-2.59; ORRo = 7.91, 95% CI: 4.93-12.68) and those with acceptable (ORRo = 4.08, 95% CI: 2.88-5.79) or poor financial conditions (ORHu = 2.05, 95% CI: 1.35-3.12; ORRo = 4.25, 95% CI: 2.52-7.15). None of the socioeconomic factors was associated with leisure time physical inactivity in either study population (Table 2), so the factors influencing physical inactivity were not analysed in a multivariate model.

**Table 2:** Univariate associations of health-related behaviours with demographic and socioeconomic factors

Variables	Health-related behaviour					
	
	Smoking		Unhealthy diet		Leisure time physical inactivity	
	
	Hungarians	Romanians	Hungarians	Romanians	Hungarians	Romanians
	
	OR (95% CI)	OR (95% CI)	OR (95% CI)	OR (95% CI)	OR (95% CI)	OR (95% CI)
Age (continuous)	0.96 (0.96-0.97)***	0.98 (0.97-0.98)***	1.00 (0.99-1.01)	1.04 (1.03-1.05)***	1.00 (0.99-1.01)	1.01 (0.99-1.02)
Gender						
Females	1.00	1.00	1.00	1.00	1.00	1.00
Males	2.29 (1.78-2.97)***	2.89 (2.16-3.86)***	2.22 (1.69-2.90)***	1.75 (1.27-2.40)***	1.11 (0.86-1.43)	0.87 (0.64-1.18)
Educational level						
High	1.00	1.00	1.00	1.00	1.00	1.00
Medium	1.66 (1.16-2.38)*	0.61 (0.41-0.89)	1.29 (0.92-1.82)	1.44 (0.97-2.15)	1.04 (0.73-1.48)	0.87 (0.56-1.35)
Low	1.23(0.84-1.82)	0.95 (0.64-1.42)	1.77 (1.21-2.59)**	7.91 (4.93-12.68)***	1.02 (0.69-1.48)	1.02 (0.66-1.57)
Self-perceived financial conditions						
Good	1.00	1.00	1.00	1.00	1.00	1.00
Acceptable	1.43 (0.97-2.09)	1.11 (0.81-1.52)	1.15 (0.81-1.63)	4.08 (2.88-5.79)***	1.05 (0.73-1.49)	0.71 (0.50-1.01)
Poor	3.13 (2.06-4.74)***	1.36 (0.89-2.07)	2.05 (1.35-3.12)**	4.25 (2.52-7.15)***	1.21 (0.81-1.79)	0.88 (0.55-1.42)

OR: odds ratio; CI: confidence interval; *p < 0.05; **p < 0.01; ***p < 0.001

**Table 3:** Univariate associations of obesity (BMI ≥ 30 kg/m^2^) with demographic, socio-economic factors and health-related behaviours

Variables	Hungarians	Romanians
	
	OR (95% CI)	OR (95% CI)
Age (continuous)	1.02 (1.01-1.03)***	1.03 (1.02-1.05)***
Gender		
Females	1.00	1.00
Males	1.03 (0.78-1.38)	1.01 (0.70-1.45)
Educational level		
High	1.00	1.00
Medium	2.20 (1.39-3.53)***	1.75 (0.91-3.36)
Low	2.43 (1.49-3.97)***	2.74 (1.48-5.09)**
Self-perceived financial conditions		
Good	1.00	1.00
Acceptable	1.11 (0.74-1.68)	1.17 (0.77-1.76)
Poor	1.39 (0.88-2.19)	1.11 (0.64-1.93)
Smoking status		
Non-smokers	1.00	1.00
Current smokers	0.65 (0.47-0.89)**	0.82 (0.56-1.20)
Dietary habits		
Healthy diet	1.00	1.00
Unhealthy diet	1.06 (0.77-1.45)	2.74 (1.61-4.66)***
Leisure time physical activity		
Active	1.00	1.00
Inactive	1.73 (1.29-2.32)***	0.68 (0.46-1.01)

OR: odds ratio; CI: confidence interval; BMI: body mass index

*p < 0.05; **p < 0.01; ***p < 0.001

An increased risk of obesity (Table 3) was found among the elderly (ORHu = 1.02, 95% CI: 1.01-1.03; ORRo = 1.03, 95% CI: 1.02-1.05) and those with low level of education (ORHu = 2.43, 95% CI: 1.49-3.97; ORRo = 2.74, 95% CI: 1.48-5.09) in both groups and also among medium educated Hungarian respondents (ORHu = 2.20, 95% CI: 1.39-3.53). In the univariate analysis conducted in Hungary, obese individuals compared to non-obese were more likely to be non-smokers (ORHu = 0.65, 95% CI: 0.47-0.89) and inactive in their leisure time (ORHu = 1.73, 95% CI: 1.29-2.32). Romanian subjects who followed an unhealthy diet were more likely to be obese than those following a healthy diet (ORRo = 2.74, 95% CI: 1.61-4.66) (Table 3).

Tables 4 and 5 present the results of multivariate logistic regression models. Involving all demographic and socioeconomic variables, the associations of health-related behaviours with these factors were similar to the results of the univariate analyses, except for the increased risk of smoking among those with poor financial conditions in Romania (ORRo = 1.72, 95% CI: 1.07-2.77) (Table 4). Including only demographic and socioeconomic factors into the multivariate model of obesity (Model 1), the risk of obesity was increased with age by years (ORHu = 1.02, 95% CI: 1.01-1.03; ORRo = 1.03, 95% CI: 1.02-1.05) both in Hungarians and Romanians and was higher among those with medium educational level (ORHu = 2.09, 95% CI: 1.29-3.37) in Hungarians (Table 5). Involving health-related behaviours (Model 2), the effects of age and education were not changed. The impact of behaviours was different by nations. On the one hand, obesity was found to be positively associated with unhealthy diet only in Romanians (ORRo = 2.10, 95% CI: 1.18-3.75); on the other hand, physically inactive Hungarians were more (ORHu = 1.74, 95% CI: 1.28-2.36), whereas inactive Romanians were less (ORRo = 0.64, 95% CI: 0.42-0.96) likely to be obese than physically active people from the same country. In Hungarians, the association between smoking and obesity was attenuated after controlling for all variables.

**Table 4:** Multivariate associations of health-related behaviours with demographic and socioeconomic factors

Variables	Health-related behaviour			
	
	Smoking		Unhealthy diet	
	
	Hungarians	Romanians	Hungarians	Romanians
	
	OR (95% CI)	OR (95% CI)	OR (95% CI)	OR (95% CI)
Age (continuous)	0.96 (0.95-0.97)***	0.98 (0.97-0.99)***	0.99 (0.99-1.00)	1.02 (1.01-1.03)***
Gender				
Females	1.00	1.00	1.00	1.00
Males	2.08 (1.57-2.74)***	3.09 (2.28-4.17)***	2.19 (1.66-2.89)***	1.91 (1.35-2.72)***
Educational level				
High	1.00	1.00	1.00	1.00
Medium	1.36 (0.92-2.01)	0.97 (0.63-1.49)	1.11 (0.79-1.59)	1.01 (0.65-1.56)
Low	1.59 (1.01-2.51)*	0.76 (0.47-1.22)	1.63 (1.06-2.51)*	3.58 (2.08-6.15)***
Self-perceived financial conditions				
Good	1.00	1.00	1.00	1.00
Acceptable	1.60 (1.06-2.42)*	1.32 (0.93-1.87)	1.21 (0.85-1.74)	3.04 (2.07-4.46)***
Poor	3.70 (2.32-5.89)***	1.72 (1.07-2.77)*	1.90 (1.22-2.96)**	3.04 (1.72-5.38)***

OR: odds ratio; CI: confidence interval; *p < 0.05; **p < 0.01; ***p < 0.001

**Table 5:** Multivariate associations of obesity (BMI ≥ 30 kg/m^2^) with demographic, socioeconomic factors and health-related behaviours

Variables	Model 1		Model 2	
	
	Hungarians	Romanians	Hungarians	Romanians
	
	OR (95% CI)	OR (95% CI)	OR (95% CI)	OR (95% CI)
Age (continuous)	1.02 (1.01-1.03)***	1.03 (1.02-1.05)***	1.02 (1.01-1.03)***	1.03 (1.02;1.04)***
Gender				
Females	1.00	1.00	1.00	1.00
Males	1.10 (0.81-1.47)	1.01 (0.69-1.46)	1.15 (0.84-1.56)	0.93 (0.63-1.38)
Educational level				
High	1.00	1.00	1.00	1.00
Medium	2.09 (1.29-3.37)**	1.53 (0.78-3.01)	2.16 (1.33-3.50)**	1.47 (0.74-2.89)
Low	1.57 (0.92-2.68)	1.55 (0.77-3.13)	1.66 (0.97-2.84)	1.35 (0.66-2.76)
Self-perceived financial conditions				
Good	1.00	1.00	1.00	1.00
Acceptable	1.02 (0.67-1.57)	0.89 (0.57-1.39)	1.04 (0.67-1.59)	0.78 (0.49-1.23)
Poor	1.25 (0.78-2.02)	0.88 (0.49-1.60)	1.31 (0.81-2.14)	0.79 (0.43-1.45)
Smoking status				
Non-smokers			1.00	1.00
Current smokers			0.70 (0.49-1.00)	1.03 (0.67-1.57)
Dietary habits				
Healthy diet			1.00	1.00
Unhealthy diet			0.99 (0.71-1.38)	2.10 (1.18-3.75)*
Leisure time physical activity				
Active			1.00	1.00
Inactive			1.74 (1.28-2.36)***	0.64 (0.42-0.96)*

BMI: body mass index; OR: odds ratio; CI: confidence interval; *p < 0.05; **p < 0.01; ***p < 0.001

Covariates in Model 1: age, gender, educational level and self-perceived financial conditions.

Covariates in Model 2: age, gender, educational level, self-perceived financial conditions, smoking status, dietary habits and leisure time physical activity.

## Discussion

Our study's aim was to describe the socioeconomic inequalities in health-related behaviours and in nutritional status of Hungarian and Romanian citizens living on both sides of the border.

Smoking prevalence was at similar level both among Hungarians (33.2%) and Romanians (36.4%) in the present study, which is in agreement with the findings from the most recent Eurobarometer study on tobacco [28]. According to that survey, the prevalence of smokers (including daily and occasionally smokers) is the highest in Greece (42%), followed by Bulgaria (38%), Latvia (37%), Romania and Hungary (both 36%).

Our study demonstrated an association of smoking with socioeconomic status, such as low education and poorer financial conditions in Hungarians, and only with poor financial conditions in Romanians. Similar associations have been found by other studies, i.e. the risk of smoking is higher in low educated and poorer people [33-35]. According to Wardle et al., cigarette smoking is more prevalent in lower social class respondents aged over 35 years [36]. An investigation on 12 European countries (around 1990) has also revealed that smoking is more prevalent among the lower educated, particularly in Northern European countries [3]. Socioeconomic inequalities in tobacco smoking are also revealed during the three periods of the Australian National Health Survey, e.g. males with the highest SES have been more likely to be never smokers than those with lower SES [7]. In a Polish study, men with higher education (aged 18 to 66 years) are less likely to smoke compared to the less educated, whereas among women with higher education smoking is more common [4]. Health interview surveys in Hungary also demonstrate that cigarette smoking is more prevalent among low educated and poorer people [37,38].

The SES related variations in the prevalence of smoking could be influenced by the actual stage of smoking epidemic in a given country [3,39]. Many countries in Eastern Europe such as Romania and Hungary are currently at stage 3 of the tobacco epidemic characterized by a marked downturn in smoking prevalence in men, a more gradual decline in women, especially in those with a higher educational level [3,9,39,40].

In our study, the prevalence of unhealthy diet was higher in Romanians than in Hungarians. The low intake of fruits and vegetables was also found in Hungary by the "National Health Interview Survey" [29]. An overview of the health status of Romanians reports that low fruit and vegetable intake is one of the leading risk factors of non-communicable diseases [23]. A study in Transylvania has found that vegetable intake is under reference values in females [41], and another study delivered in 25-65-year old subjects has found higher prevalence of unhealthy diet in men [42].

In agreement with other studies, we revealed an association of unhealthy diet with education and financial conditions in both countries. Wardle et al. have also found that low fruit and vegetable intake is more prevalent in lower social class respondents [36]. Johansson et al. have reported that social status measured by education and aggregates of SES (blue-collar and white-collar workers and income per year) is correlated to indicators of healthy diet (e.g. fruit and vegetable consumption) in men and women aged 16-79 years, e.g. those having at least 13 years of education have higher intakes of fruits, vegetables and fibre than those with less than 13 years of education [13]. Likewise, in a review paper about food patterns in terms of various socioeconomic indicators across Europe, it is stated that those who are poorer in material or social conditions are likely to follow a less healthy diet, i.e. people with lower SES consume nutrients from a less diverse food base: they eat monotonous diets with little variety [12].

In our study, leisure time physical inactivity of the participants was independent of their demographic and socioeconomic data in both countries - the result being in contrast with several reports in the literature. Haenle et al. have highlighted gender and age related differences in German adults aged 18-65 years; males are more likely than females to engage in more intense leisure time physical activity, and females in the youngest age group are the least physically active [43]. A review of several studies describes that those with higher education levels or the self-employed are more likely to be moderately active in their leisure time [12]. The Hungarian national survey (2000) has described that higher educated and wealthier people are less likely to be physically inactive in their leisure time and/or at work [32], while the next survey (2003) has found an association between financial conditions and inactivity only in females [38]. An Australian survey points at strong socioeconomic inequalities in terms of leisure time physical activity in both males and females [7], however, no associations have been found between physical activity levels in leisure time and social status indices such as the level of education and annual income in the ATTICA study in Greece among 20-89 years old persons [11].

A systematic review on the prevalence of obesity indicates geographic variations with rates being higher in Central, Eastern, and Southern Europe than in Western and Northern Europe. This geographic pattern can be explained, at least partly, by different socioeconomic conditions as well as by lifestyle and nutritional factors, but may also be partly due to ethnic differences [44]. The percentage of people who are overweight and obese reflects socioeconomic inequalities in Australia [7]. In a study on males (53-75 years) in Denmark, leisure time physical activity is associated with obesity and social class [45]. In our study, the prevalence of obesity was higher among Hungarians (22.0%) than Romanians (16.5%). The risk of obesity was higher in older people in both nations, and was associated with medium and low educational levels in Hungarians.

Obesity, associated with lifestyle and characterized by unbalanced diets high in calories and also by inadequate physical activity, is considered as a risk factor for numerous diseases [46]. In our study, a positive association was found between leisure time physical inactivity and obesity in Hungarians, whereas a negative association was revealed in Romanians. The association between physical inactivity and obesity in Hungarians was in agreement with the results of FINRISK cross-sectional studies in the 25-64-years old population: leisure-time physical activity was inversely associated with obesity both in men and women [15]. The negative association between leisure time physical inactivity and obesity in Romanians was inconsistent with most of the previous results. The findings of a prospective cohort study suggests, however, that high BMI is a determinant of sedentary lifestyle, but it has failed to provide unambiguous evidence for an effect of sedentary lifestyle on weight gain [47]. This seemingly ambiguous result might be due to the fact that we examined only leisure time physical activity and did not cover activity during work. Those being engaged in strenuous physical activity during work are more probably inactive in their leisure time than those having a sedentary occupation. It should also be mentioned that the comparison of our physical activity related results with the findings of previous studies was slightly limited because the measurement for leisure time physical activity has not been previously validated.

In Romanians, unhealthy diet was associated with the risk of obesity, though no association was found in Hungarians. Our findings in Romanians are also in line with the results of FINRISK studies showing that obese subjects appear to consume less fruits and vegetables [15], and with the results of a cross-sectional study delivered in Romanian primary care settings in Iasi where obesity is more prevalent in case of unhealthy diet in males [42].

No association was found between smoking and obesity in our study that is in contrast with several other reports. The study delivered in Iasi (Romania) has described higher rates of obesity among male smokers [42]. The FINRISK study has highlighted that ex-smokers are heavier than non-smokers both among men and women [15]. The Copenhagen male study reveals that leisure time physical activity and smoking habits are associated with obesity [45]. The results of a cross-sectional study in 18-75 years show that smoking status, educational level, time spent in health related sport activities and sedentary behaviour are associated with the likelihood of being overweight [16].

A healthy lifestyle, with its behavioural emphasis might not depend only on the individuals' decisions. Individuals can make choices in a social context [48], and helping individuals to change unhealthy behaviour should always be part of the health promotion. Our study may call the attention to the inequalities in smoking and dietary habits in relation to the socioeconomic status: the occurrence of health-damaging behaviours was more common among the less educated people in both countries. Concerning obesity, we found different situations in the two countries: the effect of dietary habits was detected only in Romanians, and the role of the physical inactivity was dissimilar.

The fact that the emerging health and health-related problems on both sides of the border and their socioeconomic background have common characteristics may draw our attention to the importance of seeking for mutual solutions. These might be realised in the framework of cross-border community based health promotion programmes that would be supported by the European Union, thus, the common problems occurring in the regions near the border could be solved together. Our study may provide some practical implications for formulating programmes that are aimed at improving the healthy behaviour in Hungarians and Romanians. Our results may point out the need for developing interventional strategies, focusing more on people in lower socioeconomic status, in order to reduce the existing inequalities in health and health-related behaviours.

In interpreting our results, it might be important to keep in mind the study's limitations. Our data were obtained in cross-sectional surveys where socioeconomic characteristics were asked simultaneously with the health-related behaviours. Strict causal interpretations should therefore be avoided. Data from self-reports tend to be inaccurate in some instances, e.g. self-reported weight and height may underestimate the prevalence of obesity. Socioeconomic status was measured according to the educational level and self-perceived financial condition, similarly to but not by the same measures that have been used in other studies. Health-related behaviours were measured by simple questions. One part of the measures, such as the measurement of leisure time physical activity was used primarily. One of the limitations of our study was the crude assessment of leisure time physical activity by questions without previous validation with the risk of misclassification. Our categories indexing the physical activity should be regarded as reflecting on common patterns of leisure time physical activity through one year, rather than precise measures of levels. They mainly inform us rather about the prevalence of leisure time physical inactivity than the measure of the level of activity. Another limitation of our study concerning the results on physical activity was that we examined only leisure time physical activity and not activity during work. However, the questions concerning diet and smoking were used according to previous Hungarian and international research in population based epidemiological studies (see the Methods), thus, results can be compared with data published by other researchers and can also be generalized. It may be that the applied measures seem to be too general, but it allows for the feasible involvement of people with various ages, educational levels, etc. Despite these limitations, this study might provide a relevant picture on the prevalence of health-related behaviours and obesity in relation to socioeconomic factors both in Hungarians and Romanians.

## Conclusion

In conclusion, the present study shows that socioeconomic status might be associated with health-related behaviours in a small area of Hungary and Romania. The results may suggest that the socioeconomic inequalities should be taken into account when planning health promotional policies or intervention programmes in the cross-border population.

## List of abbreviations

BMI: body mass index; CI: confidence interval; EU: European Union; Hu: Hungarian; OR: odds ratio; Ro: Romanian; SD: standard deviation; SES: socioeconomic status

## Competing interests

The authors declare that they have no competing interests.

## Authors' contributions

EN coordinated the survey, was responsible for data collection and data entry and drafted the manuscript. EP designed the study, performed statistical analysis and drafted the manuscript. Both authors read and approved the final manuscript.

## Pre-publication history

The pre-publication history for this paper can be accessed here:

http://www.biomedcentral.com/1471-2458/12/60/prepub
